# Photon extraction enhancement of praseodymium ions in gallium nitride nanopillars

**DOI:** 10.1038/s41598-022-25522-6

**Published:** 2022-12-08

**Authors:** Shin-ichiro Sato, Shuo Li, Andrew D. Greentree, Manato Deki, Tomoaki Nishimura, Hirotaka Watanabe, Shugo Nitta, Yoshio Honda, Hiroshi Amano, Brant C. Gibson, Takeshi Ohshima

**Affiliations:** 1 Quantum Beam Science Research Directorate, National Institutes for Quantum Science and Technology, 1233 Watanuki, Takasaki, Gunma 370-1292 Japan; 2 Australian Research Council Centre of Excellence for Nanoscale BioPhotonics, RMIT University, Melbourne, VIC 3001 Australia; 3 Venture Business Laboratory, Nagoya University, Furo-Cho, Chikusa-Ku, Nagoya, Nagoya 464-8601 Japan; 4 Research Center of Ion Beam Technology, Hosei University, 3-7-2 Kajino-Cho, Koganei, Tokyo 184-8584 Japan; 5 Institute of Materials and Systems for Sustainability, Nagoya University, Furo-Cho, Chikusa-Ku, Nagoya, 464-8601 Japan

**Keywords:** Lasers, LEDs and light sources, Optical materials and structures, Materials for optics, Nanoscale materials, Nanophotonics and plasmonics

## Abstract

Lanthanoid-doped Gallium Nitride (GaN) integrated into nanophotonic technologies is a promising candidate for room-temperature quantum photon sources for quantum technology applications. We manufactured praseodymium (Pr)-doped GaN nanopillars of varying size, and showed significantly enhanced room-temperature photon extraction efficiency compared to unstructured Pr-doped GaN. Implanted Pr ions in GaN show two main emission peaks at 650.3 nm and 651.8 nm which are attributed to ^3^*P*_0_-^3^*F*_2_ transition in the 4*f*-shell. The maximum observed enhancement ratio was 23.5 for 200 nm diameter circular pillars, which can be divided into the emitted photon extraction enhancement by a factor of 4.5 and the photon collection enhancement by a factor of 5.2. The enhancement mechanism is explained by the eigenmode resonance inside the nanopillar. Our study provides a pathway for Lanthanoid-doped GaN nano/micro-scale photon emitters and quantum technology applications.

## Introduction

Efficient and ecological small photon emitters, e.g. nano/micro-scale light emitting diode (LED)^1–6^ and monolithic RGB emitters^7–9^, are required for next generation lighting and display applications. Reliable single photon sources (SPS or single photon emitter: SPE)^10^, which provide on-demand single photons are indispensable in the field of rapidly growing quantum technologies such as quantum computing, quantum sensing, and quantum communications (quantum internet)^11–14^. For maximum utility, these sources should be room temperature, compatible with mature fabrication processes, and able to be integrated with other photonic systems. These demands have stimulated studies on gallium nitride (GaN)-based photon emitters and devices, which is a promising photonic platform.

Lanthanoid (Ln)-doped GaN (Ln:GaN) exhibits visible/near infrared (VIS–NIR) emission that is temperature insensitive, sharp and stable, since the energy levels in 4*f*-shell which are involved in the luminescence transitions are surrounded by filled 5*s* and 5*p* orbitals and thus isolated from free carriers in the host material. Additionally, such materials utilize well-developed GaN platform, enabling integration into more complex devices^15^. The photon emission can be electrically controlled and Ln:GaN LEDs have been demonstrated^16–22^. These superior optical and opto-electronic properties are also suitable for SPS applications. Single photon emission from single praseodymium (Pr) ions implanted in a YAG crystal at room temperature (RT)^23^ and coherent optical and spin control of single Pr ions have been reported^24–27^. Although these previous literatures have demonstrated the strong potential of Pr-SPS, insulating materials have been used for the host and the electric control of photon emissions has not been considered. In contrast, electrically controlled SPS operating at RT, which is particularly advantageous for practical applications, is feasible by Pr:GaN. However, there is still a significant limitation resulting from a poor light extraction efficiency due to the high refractive index of GaN (*n*_GaN_ = 2.3). Therefore, improvement of the photon extraction efficiency is crucial for the realization of reliable Pr:GaN SPS.

An important method for improving photon extraction efficiency is through the creation of surface nanostructures, such as waveguides, cavities, and other resonant structures. Such structures can be used to improve photon extraction efficiency, photon emission rate, and internal quantum efficiency of the photon emitters^28^. Nanostructure fabrication can be described as bottom-up or top-down. With respect to the bottom-up approach, selective-area growth of nanocolumns with SiN_*x*_ or Ti masks is employed. Obtained nanocolumns exhibit dislocation-free and strain relaxation properties^8,9,29–33^. With respect to the top-down approach, lithography and dry/wet etching techniques are mainly used and highly flexible nanostructures are available^2,3,34,35^. Because of its structural flexibility, the top-down is more advantageous than the bottom-up in terms of integration of luminescent centers into nanostructures. By embedding quantum dots/wells into (In)GaN nanocolumns (or nanopillars, nanorods), significant enhancements of photon extraction efficiency and internal quantum efficiency have been reported^35^, and also, the feasibility for SPS applications have been demonstrated^36–39^.

On the other hand, little is known about the optical coupling of Ln ions to GaN nanostructures and its effectiveness with respect to photon extraction enhancement and photon emission rate, despite the fact that studies on Ln:GaN materials and devices have been conducted for more than two decades. Although previous work has reported Ln-related emission from a nanopatterned GaN^40^ and its enhancement^16,41^, the optimization of nanostructures based on the enhancement mechanism has not been performed. One of the greatest advantages of Ln:GaN is the wavelength tunability from VIS to NIR by appropriate choice of Ln elements. Such dopant-dependent wavelength variation is unavailable in InGaN quantum structures. Nanostructures to improve the photon extraction efficiency strongly depend on both the photon emission mechanism and wavelength, and thus some degree of optimization is required for each class of Ln:GaN device.

Here we discuss optical properties of praseodymium (Pr)-implanted GaN nanopillars of varying size and structure, which show different photon extraction enhancement. These nanopillars are fabricated using the top-down approach: electron beam lithography, metal deposition, and dry etching techniques. Room temperature (RT) optical properties of individual nanopillars with different sizes are characterized by using a home-built confocal microscopy. The photon emission saturation shows that both the excitation laser collection and the extraction efficiency of emitted photons from implanted-Pr ions are enhanced by nanopillar structures when compared to the photon emission at a region where no pillars are etched into the structure. To theoretically understand the effects of pillar size and structure, we also establish the model for the photon emission enhancement of GaN nanopillars with different sizes based on the eigenmode analysis, and the theoretical results verifies the measured emission enhancement in experiment.

## Experimental

**Figure 1:**
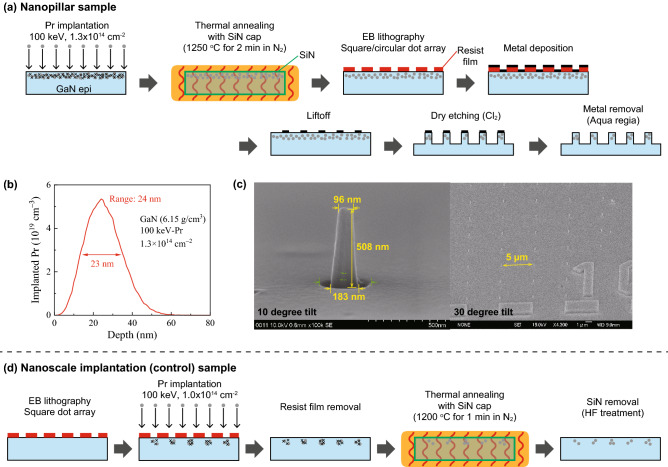
(**a**) Fabrication procedure for the nanopillar sample. The detail is described in the main text. (**b**) Depth profile of implanted 100 keV-Pr ions in GaN at the fluence of 1.3 × 10^14^ cm^−2^, calculated by the Monte Carlo simulation code, TRIM. (**c**) Representative SEM images of a circular pillar with the diameter of 100 nm (left) and the pillar array (right). The grid interval for 100 nm circular pillar is 5 µm. (**d**) Fabrication procedure for the nanoscale implantation (control) sample.

The fabrication procedure of Pr-doped GaN nanopillars is summarized in Fig. 1a. Nanopillar structured surface was formed on an undoped GaN epilayer with thickness of 15 μm grown on n-type GaN (n-GaN) which were implanted with 100 keV-Pr at a fluence of 1.3 × 10^14^ cm^−2^ at RT. The ion implantation was performed at Takasaki Advanced Radiation Research Institute, National Institutes for Quantum Science and Technology (QST). The peak implanted region and Pr concentration were estimated to be 24 nm from the top of pillars and 5.3 × 10^19^ cm^−3^, respectively, according to the Monte Carlo simulation code TRIM (Fig. 1b)^42^. After Pr ion implantation, the sample was annealed at 1250 °C for 2 min under N_2_ atmosphere (1 atm) using an infrared furnace to remove radiation induced defects and to activate implanted Pr ions as luminescence centers. Prior to the thermal annealing treatment, a 50 nm thick SiN cap layer was formed on the surface using a magnetron sputtering method to suppress the deterioration of crystallinity by preferential evaporation of nitrogen^43^. The furnace temperature rose to the set temperature in 1 min, then kept at the temperature for set time, and was then naturally cooled down to RT for about 15 min. The SiN cap layer on the samples were subsequently removed by hydrofluoric acid treatment (HF:H_2_O = 1:5, 20 min). Resist film was coated on the GaN epilayer and dot arrays (squares and circles) were formed using electron beam (EB) lithography. The pillar size (side length for squares and diameters for circles) ranged from 100 nm to 2 µm, and the grid pitch of dot arrays was 5 µm or 10 µm. A 120 nm thick Ni layer was then deposited on the sample using electron beam evaporation. Following lift-off process, metal dot arrays are formed on the sample surface. These arrays were used as a mask for etching the GaN epilayer by ICP (Inductively Coupled Plasma) dry etching operating at 2.0 Pa with a Cl_2_ 30 sccm. The ICP and bias powers were 150 W and 30 W. Finally, the metal layer was removed by acid treatment (aqua regia). The fabricated nanopillar structures was investigated by scanning electron microscopy (SEM) as shown in Fig. 1c. All pillars were fabricated mostly within the error of 5%. The pillar length and taper angle were measured to be 510 nm and 4 degrees, which were constant with pillar size. Also, a micro-trench structure was found around the bottom of nanopillars, indicating the dry etching conditions could be further improved for better fabrication, although we do not believe that the micro-trench was significant for our results. A sample implanted into nanoscale square regions was also fabricated for comparison, as shown in Fig. 1d (control sample). The EB lithographic pattern was formed on the resist film and then 100 keV Pr ions were implanted at RT. The implantation fluence was 1.0 × 10^14^ cm^−2^. The resist film was removed after implantation and the thermal annealing at 1200 °C for 1 min was performed. The side length of square implantation area ranged from 100 nm to 2 µm. Detailed optical properties of the nanoscale implantation samples have been reported elsewhere^44,45^.

Photon emission properties of the samples were characterized by a home-built laser scanning confocal microscopy (CFM). A Supercontinuum wavelength-tunable pulsed laser (6 ps pulse width, 80 MHz repetition rate) was used for excitation. Photon emission from the Pr-implanted regions was collected with an objective lens (numerical aperture, NA = 0.90) and detected by a Si avalanche photo-diode (APD). The PL spectra were investigated by an imaging spectrometer installed in the CFM. A 650 nm band pass filter (13 nm bandwidth) was placed in front of the APD and the spectrometer to selectively collect photons emitted from the implanted-Pr ions. The PLE spectra ranging from 400 to 610 nm and the luminescence lifetime were also measured using the same measurement setup. For the luminescence lifetime measurement, the repetition rate of pulsed laser was set to be 0.1 MHz. All measurements were performed at RT.

## Results and discussion

### Optical properties of Pr ions in different GaN nanopillars

**Figure 2:**
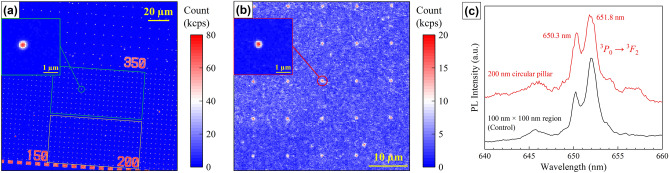
(**a**) A CFM image of the Pr-doped GaN nanopillars. Squares in green and yellow denote the 200 nm circular and square pillar arrays with 5 μm interval, respectively. The inset shows the zoom-in CFM image of a 200 nm circular pillar. The excitation laser wavelength and power were 525 nm and 20 µW, respectively. (**b**) A CFM image of the nanoscale implantation (control) sample. 100 × 100 nm square implanted region arrays with 10 µm interval are shown. The inset shows the zoom-in CFM image of a square implanted region. The excitation laser wavelength and power were 525 nm and 0.5 mW (51 µW in the inset), respectively. (**c**) PL spectra f the Pr-doped GaN circular pillar with 200 nm in diameter and 100 × 100 nm square implanted region.

A representative CFM image of the Pr-doped GaN nanopillars is shown in Fig. 2a. The obtained CFM image reproduced the nanopillar array patterns. The inset shows the zoom-in CFM image of a 200 nm circular pillar. The observed luminescence spot was larger than the actual size of nanopillar and the image reflected the CFM point spread function (PSF), since the lateral resolution (356 nm according to the Rayleigh limit) of CFM was larger than the 200 nm circular pillar^44^. Figure 2b shows a CFM image of the nanoscale implantation (control) sample. The implantation area was 100 × 100 nm and the grid interval was 10 µm. The obtained CFM image reproduced the designed EB lithography pattern, indicating the EB lithography pattern was successfully transferred to the Pr implantation pattern. In both CFM images, the implanted-Pr ions were excited with 525 nm laser for the resonant excitation. We revealed two resonant excitation peaks at 506 nm (2.45 eV) and 525 nm (2.36 eV) which are presumably due to the ^3^*H*_4_-^3^*P*_1_ transition^22,46^ from the analysis of photoluminescence excitation spectrum (see Supplementary Information for more detail). PL spectrum of the 200 nm circular pillar was also characterized as shown in Fig. 2c. Two emission peaks at 650.3 nm and 651.8 nm are attributed to ^3^*P*_0_-^3^*F*_2_ transition in the 4*f*-shell of Pr^3+^ (trivalent Pr) ions and the multiple peaks are caused by crystal field splitting^47^. A similar PL spectrum was obtained from the control sample, indicating that the nanostructuring does not affect the PL spectrum.

**Figure 3:**
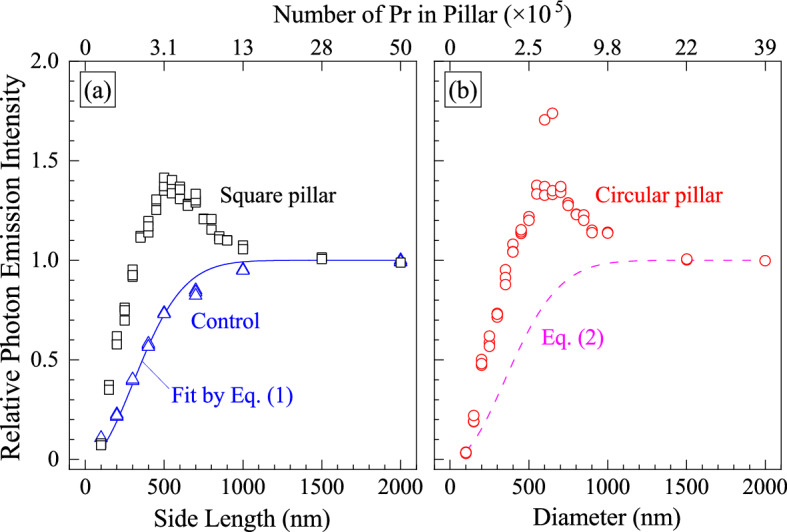
Photon emission intensity as a function of pillar size and number of Pr in pillars: (**a**) square pillars and (**b**) circular pillars. The excitation wavelength and power were 525 nm and 13.47 kW/cm^2^ (100 µW), respectively. In (**a**), the intensity for square implanted regions in the control sample and a fitting curve by Eq. (1) are also shown for comparison (open triangles and line in blue). In (**b**), a modeled data by Eq. (2) is also drawn as a pink dashed line. The ordinate is normalized by the value at a uniformly implanted region in the samples.

Figure 3 shows the photon emission intensity from Pr ions with different pillar sizes. The data for implanted-Pr ions without pillar structures (control sample) are also shown as blue triangles for comparison. The ordinate is normalized by the intensity at a uniformly implanted region of the sample. The excitation laser was focused on the center of pillars or implantation regions, and the average photon counts for 30 s were recorded. The photon counts from implanted regions were stable for a prolonged time, with no observed photobleaching. The background counts, which was measured at an unimplanted region, was subtracted to obtain the net photon emission intensity from implanted-Pr ions (2.3 kcps for the nanopillar sample and 1.0 kcps for the nanoscale implantation sample). In the case of nanoscale implantation sample, the measured photon emission intensity increases with increasing size of implantation region as the number of Pr ions excited increased. Note that the area density of implanted Pr ions was constant (1.0 × 10^14^ cm^−2^). The photon counts become constant when the implantation region is far larger than the excitation laser spot. Assuming that the PSF is Gaussian, the fraction of activated implanted-Pr ions is unchanged regardless of implantation area, and the photon emission intensity is proportional to the laser intensity. In this limit, the relationship between the photon emission intensity (*I*) and the side length of square implanted region (*d*) can be expressed as^44^:

1 $$\begin{array}{*{20}c} {I\left( d \right) \propto {\text{erf}}^{ 2} \left( {\frac{d}{2\sqrt 2 s}} \right)} \\ \end{array}$$

where *s* is the standard deviation of the Gaussian. Also, when the implantation region is circular shape, the photon emission intensity is derived as its diameter (*R*) in the same fashion:

2 $$\begin{array}{*{20}c} {I\left( R \right) \propto 1 - {\text{exp}}\left( { - \frac{{R^{2} }}{{8s^{2} }}} \right)} \\ \end{array}$$

The calculation result of Eq. (1), shown as a solid blue line in Fig. 3a, is well fitted to experimental data for the square implantation regions (open blue triangle) and we obtain *s* = 172 nm. The FWHM ($2\sqrt {2\ln \left( 2 \right)} s$. ) is calculated to be 405 nm, being slightly larger than the Rayleigh limit (356 nm). This is thought to be due to misalignment of the confocal system. A curve by Eq. (2) when *s* = 172 nm is drawn as a pink dashed line in Fig. 3b. In both cases, the photon emission intensity monotonically decreased with decreasing size of implantation regions (side length and diameter). On the other hand, in the case of nanopillar samples, the photon emission intensity never followed the Eqs. (1) and (2), and the photon emission intensity was the highest when the pillar size was around 500–650 nm. In addition, it was found that the pillar size where the highest intensity was obtained depended on the excitation laser intensity, which will be discussed in the next paragraph. These rests cannot be interpreted by the above explanation based on the Eqs. (1) and (2), and indicate that the photon emission rate and/or the photon extraction efficiency was enhanced by the nanopillar structures. In the case of > 1 µm pillars, little enhancement appeared, and this is thought to be related to the net laser spot diameter, which will be discussed in the next section.

**Figure 4:**
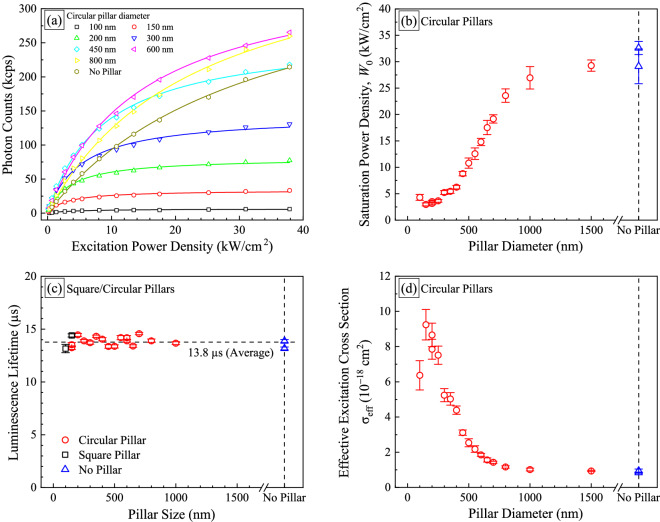
(**a**) Photon emission intensity as a function of excitation power density with different circular pillar diameters ranging from 100 to 800 nm. Photon emission intensity at a control region where no pillars were etched into the structure is also shown as closed gold hexagons for comparison. (**b**) Saturation power density *W*_0_ as a function of pillar diameters. The data for control regions are shown as blue triangles. (**c**) Luminescence lifetime as a function of pillar diameters. (**d**) Effective excitation cross section (σ_eff_) as a function of pillar diameters. Error bars in (**b**–**d**) denote the standard deviation of the data.

To explore photon emission properties in < 1 µm pillars where emission enhancement appeared, excitation laser power dependence and luminescence lifetime were investigated. Figure 4 shows the excitation laser power dependence of photon emission intensity from circular pillar with different diameters. The data obtained from a control region where no pillars were etched into the structure is also shown for comparison as closed gold hexagons. The ordinate is the photon counts from implanted-Pr ions with the background subtracted. In all cases, the photon emission intensity increased with increasing excitation power density and showed the saturation behavior. The saturation behavior of photon emission intensity can be expressed as:

3 $$\begin{array}{*{20}c} {I \propto \left( {1 + \frac{{W_{0} }}{W}} \right)^{ - 1} } \\ \end{array}$$

where *I*, *W*, and *W*_0_ are the photon counts from Pr^3+^, the laser power density, and the saturation power density, respectively^15,44^. The calculated results, shown as solid lines in Fig. 4(a, are well fitted to the experimental data. When comparing to the data for control region, the 600 nm pillars showed the hhest photon emission intensity regardless of excitation power density and the photon emission intensity was reduced with decreasing the pillar diameters at less than 600 nm. Interestingly, as the pillar diameter was reduced, the saturation of photon emission intensity appeared at lower excitation power density. In other words, the saturation power density, *W*_0_ was reduced with decreasing pillar diameters as shown in Fig. 4b.

Different values of the saturation power density are attributed to the different excitation cross sections and/or the different transition lifetimes. The saturation power density can be expressed as:

4 $$\begin{array}{*{20}c} {W_{0} = \frac{{E_{{{\text{ex}}}} }}{{\sigma_{{{\text{eff}}}} \times \tau_{1} }}} \\ \end{array}$$

where *E*_ex_, σ_eff_, τ_1_ are the excitation photon energy, the effective excitation cross section, and the luminescence lifetime, respectively. The luminescence lifetime of Pr^3+^ was characterized by the time-resolved photoluminescence (TRPL) spectroscopy on the CFM system, at a repetition rate of 0.1 MHz (the minimum rate we could access). Photon emission from Pr^3+^ ions excited by previous pulses is also included in the TRPL spectrum as the excitation pulse interval (10 µs) is comparable to (or longer than) the expected luminescence lifetime. In this case, the decay component for Pr^3+^ ions was represented by the sum of series of exponential decay function^44^:

5 $$\begin{array}{*{20}c} {\begin{array}{*{20}c} {\begin{array}{*{20}c} {I\left( t \right) = A_{1} \mathop \sum \limits_{k} \exp \left( { - \frac{{t + kt_{{\text{R}}} }}{{\tau_{1} }}} \right) + A_{2} {\text{exp}}\left( { - \frac{t}{{\tau_{2} }}} \right) + B} \\ { = A_{1} \frac{{\exp \left( { - \frac{{t + t_{{\text{R}}} }}{{\tau_{1} }}} \right)}}{{1 - \exp \left( { - \frac{{t_{{\text{R}}} }}{{\tau_{1} }}} \right)}} + A_{2} {\text{exp}}\left( { - \frac{t}{{\tau_{2} }}} \right) + B} \\ \end{array} } \\ \end{array} } \\ \end{array}$$

where *I*(*t*) is the photon counts at the time *t*, *τ*_2_ the residual defect luminescence lifetime (~ ns), *t*_R_ the excitation pulse repetition rate (10 µs), and *B* the background photon count including system noise. *A*_1_ and *A*_2_ are the fitting parameters reflecting the photon emission intensities from the Pr^3+^ ions and the residual defects, resptively. Note that the first term becomes $A_{1} {\text{exp}}\left( { - t/\tau_{1} } \right)$ when $t_{{\text{R}}} \gg \tau_{1}$. The relevance of Eq. (5) was confirmed by comparison to another conventional TRPL measurement system. Figure 4c shows the luminescence lifetime of Pr^3+^ as a function of pillar size. The luminescence lifetime was 13.8 µs on average, being independent of pillar size and structure. In other words, no significant change was found in photon emission rate from Pr ions by pillar size and structure. Small variation among the obtained values is thought to be due to slight difference in GaN crystallinity and neighboring defects in GaN nanopillars^48^.

Values of σ_eff_ can be derived from Eq. (2) using the luminescence lifetime (τ_1_) obtained from Fig. 4c. Figure 4d shows σ_eff_ as a function of circular pillar diameters. It is shown that σ_eff_ increased with decreasing pillar diameters and showed the highest value of 9 × 10^−18^ cm^2^ at the diameter of 150 ~ 200 nm, being slightly reduced at the diameter of 100 nm. The highest value is tenfold higher than the value at the control region (open blue triangles, 9 × 10^−19^ cm^2^). This result indicates that the nanopillar structure effectively collected the excitation laser and had Pr^3+^ ions excited with smaller laser power than the control region.

Here, we discuss the mechanism of photon emission enhancement due to nanopillar structures observed in Fig. 3. As shown in Fig. 4c, the photon emission rate (luminescence lifetime) of implanted Pr was unchanged by nanopillars. The unchanged photon emission rate and the PL spectrum (Fig. 2c) suggests that the nanopillar structures behaved as broadband nanoantennas and enhanced the entire photon emisssion from implanted Pr^49^. In this case, three parameters are involved into the photon emission enhancement^49,50^: the collection rate of excitation laser of nanopillars or implanted regions (*P*); the extraction efficiency of emitted photons from Pr^3+^ ions (*C*); and the quantum efficiency of Pr^3+^ ions ($QE$). The quantum effiiency is defined as $QE = k_{r} /\left( {k_{nr} + k_{r} } \right)$ where $k_{r}$ and $k_{nr}$ are the radiative and non-radiative decay rate of Pr^3+^ ions, respectively. The photon emission rate (total decay rate), $k$ is equivalent to the reciprocal of luminescence lifetime, $\tau_{1}$ (*i.e.* $k = k_{nr} + k_{r} = 1/\tau_{1}$). In addition to them, the activation rate of implanted-Pr as luminescence centers (*η*) should be considered as not all implanted Pr can activate as luminescence centers. Then, the enhancement ratio, *M* can be expressed as:

6 $$\begin{array}{*{20}c} {M = \frac{{P_{{{\text{Pillar}}}} }}{{P_{{{\text{Ct}}}} }}\;\frac{{C_{{{\text{Pillar}}}} }}{{C_{{{\text{Ct}}}} }}\;\frac{{QE_{{{\text{Pillar}}}} }}{{QE_{{{\text{Ct}}}} }}\;\frac{{\eta_{{{\text{Pillar}}}} }}{{\eta_{{{\text{Ct}}}} }}} \\ \end{array}$$

**Figure 5:**
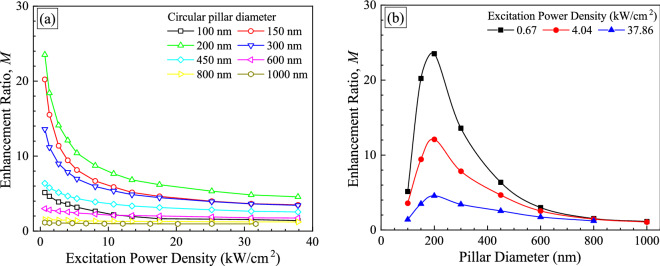
Enhancement ratio, *M* of photon emission from Pr ions in nanopillars ranging from 100 to 1000 nm in size. The same data are shown in (**a**) and (**b**), but the ordinate is the excitation power density in (**a**) and the pillar diameter in (**b**). Only three data set for low (0.67 kW/cm^2^), middle (4.04 kW/cm^2^), and high (37.86 kW/cm^2^) excitation power densities are shown in (**b**).

The subscripts “Pillar” and “Ct” denote the values for nanopillar and control (unstructured) region. The activation rate, *η* and the quantum efficiency, $QE$ are thought to be independent of nanopillar structure (*i.e*., $\eta_{{{\text{Pillar}}}} = \eta_{{{\text{Ct}}}}$ and $QE_{{{\text{Pillar}}}} = QE_{{{\text{Ct}}}}$ ), although their derivation is beyond the scope of this study. Hence, the result of Fig. 3 is attributed to the enhancement of photon collection rate and extraction efficiency of emitted photons. Then, we can simplify the Eq. (6) as follows:

7 $$\begin{array}{*{20}c} {M\left( {R,W} \right) = \frac{{P_{{{\text{Pillar}}}} }}{{P_{{{\text{Ct}}}} }}\;\frac{{C_{{{\text{Pillar}}}} }}{{C_{{{\text{Ct}}}} }} = \frac{{Y\left( {R, W} \right)}}{{Y_{{{\text{Ct}}}} }}} \\ \end{array}$$

where, *Y* is the photon emission intensity per implanted Pr. *Y* is defined as $Y\left( {R,W} \right) = I\left( {R,W} \right)/N_{{{\text{Pr}}}}$. , where *N*_Pr_ is the number of Pr ions in nanopillars or implanted regions excited by the laser. When the laser spot diameter is larger than the nanopillars and the implanted regions, *N*_Pr_ is the function of the pillar diameters (*R*) and the laser power density (*W*). The enhancement ratio as a function of laser power density with different circular pillar diameters ishown in Fig. 5a and b. Note that the ordinate in Fig. 5 is different from that in Fig. 3, since the enhancement ratio is defined as PL intensity ratio with single Pr ion in pillar to single Pr ion in control region. It can be seen from Fig. 5a that the enhancement ratio decreased with increasing laser power density regardless of pillar diameters. This is thought to be because the enhancement of excitaiton laser collection is lower as the excitation laser density is higher. As shown in Fig. 5b, the highest enhancement ratio was obtained at the 200 nm circular pillar and the maximum value was 23.5 when the laser power density was the lowest (0.67 kW/cm^2^). This value is higher than the value which has previously reported by Lesage et al.^41^. They have reported that photon extraction efficiency from Eu^3+^ ions in GaN, showing the photon emission at ~ 621 nm, increased up to 12.8 times due to a nano-patterned structure. The maximum enhancement ratio for extraction efficiency of emitted photons is estimated to be 4.5 in the limit as the excitation power density approaches the inifinity in Fig. 5a, and thus, the maximum enhancement ratio for photon collection is estimated to be 5.2 at the lowest laser power density.

### Optical properties of praseodymium ions in different GaN nanopillars

In this section, we study the enhancement mechanism theoretically and analyze the factors that contribute to the enhancement. The theoretical analysis is based on the hypothesis that the whole enhancement curve shown in Fig. 3 is a combination of: excitation enhancement, emission enhancement, implanted area function shown as the emission intensity at a region where no pillars were etched into the structure (Eqs. 1 and 2), and the objective numerical aperture. We identify three regimes for enhancement, based on pillar size:

(i) when the pillar size is < 200 nm, the implanted area function is the dominant factor. The pillar size is smaller than the free-space excitation wavelength, λ_ex_ = 525.5 nm, and the excitation wavelength in the pillar, $\lambda_{{{\text{ex}}}} /n_{{{\text{GaN}}}} = {228}{\text{.5}}$ nm; as well as the emission length λ_em_ = 652 nm. In this regime, neither the excitation nor emission wavelength are able to generate strong resonance inside the pillar. Consequently, the experimental data in Fig. 3 shows little intensity difference between the nanopillar and control scenario (see the data at side length = 100 nm in (a)).
(ii) when the pillar size is between 200 nm ~ 1 μm diameter, the pillar resonance is the dominated mechanism. In this region, both excitation and emission wavelength can excite resonant modes of the pillar in both transverse direction (across the pillar cross-section) and longitudinal direction (along the pillar height). A series of eigenmodes will then contribute to the intensity enhancement in the nanopillar.
(iii) when pillar size is > 1 μm, the pillar top is larger than the objective focal spot size, also much larger than the pillar height, since the net laser spot diameter is estimated to be *D* = 712 nm (*D* = 1.22 × λ_ex_ / NA) in the experiment. The detected intensity is limited by the objective NA and the pillar geometry is close to a platform, whereas the resonance effect inside the pillar is diminishing. Therefore, the measured intensity is no longer enhanced and approaching to the no-pillar intensity plateau in Fig. 3 curves.

**Figure 6:**
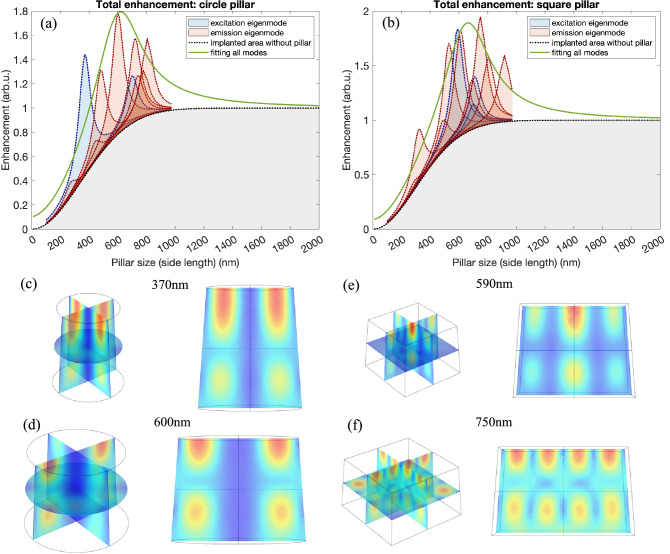
(**a**) Circular pillar enhancement simulation and (**b**) square pillar enhancement simulation. The blue and red peaks show the contribution of excitation and emission eigenmodes, respectively, and the gray area shows the contribution from nanoscale implantation intensity, *I*_0_. A Lorentz curve (green) centered 600 nm with FWHM 200 nm is used as the ultimate fitting to show the enhancement envelop. Eigenmode patterns at maximum enhancement size are illustrated in (**c**–**f**). The left column is the 3D view of the eigenmodes, and the right column shows the view of eigenmode patterns from the pillar side, which clearly present the feature of standing waves. Circular pillar size of max excitation: 370 nm, size of max emission: 600 nm. Square pillar size of max excitation: 590 nm, size of max emission: 750 nm.

To simulate these scenarios, we built an eigenmode model with finite element method (FEM) (COMSOL Multiphysics) and theoretical fitting model using MATLAB. The 3D geometry is shown in Fig. 6c–f. The model computes both GaN (*n*_GaN_ = 2.3) circular and square pillars with the height 510 nm and size *a*, where *a* is the circle diameter/the square side length of pillar top. Both the circular and square pillars have 4 degree tapered side walls so the pillar bottom is about 80 nm larger than the top. The bottom of the pillars is set as scattering boundary condition to simulate the leakage into the GaN substrate.

We calculated the eigenmodes at both excitation and emission wavelength regions, excitation centered at λ_ex_ = 525.5 nm with a bandwidth of 1 nm, emission centered at λ_em_ = 652 nm with a bandwidth of 5 nm. The electromagnetic fields of eigenmodes in the 3D pillar geometry are computed with the COMSOL eigenfrequency solver, and the simulation results are obtained by integral the intensity over the pillar top. Considering the focal spot diameter *D* of the objective, the integral area is the pillar top area when the pillar size is smaller than the focal spot diameter (*a* < *D*), while the integral area is a circle with diameter = *D* on the pillar top when *a* > *D*.

The simulated total enhancement of pillar intensity is shown in Fig. 6a and b, showing the circular and square pillar simulation, respectively. For each pillar size, we treat all other loss mechanisms, *i.e.* the contribution from other factors besides pillar geometry as an overall effect (and ultimately unknown) such as substrate leakage, size variation in fabrication, imperfect circle/square structures, etc. In addition, we consider that each Pr ion in a pillar is coupled to a slightly different mode from the other Pr ions depending on the location. To simulate overall effects, we use the Lorentz function expansion. Based on the enhancement contributors, we define the enhancement formula as

8 $$\begin{array}{*{20}c} {I_{{{\text{pillar}}}} = f + I_{0} } \\ \end{array}$$

9 $$\begin{array}{*{20}c} {f\left( {x; a, \delta ,h } \right) = h\frac{1}{{\delta \left[ {1 + \left( {\frac{x - a}{\delta }} \right)^{2} } \right]}}} \\ \end{array}$$

where *a* is the pillar size, δ is the half-width at half-maximum (HWHM) of Lorentz peak, and *h* is the peak height. In reality the size variation in fabrication would lead to a broader and more complex eigenmodes distribution. Considering the small variance won’t change the dominating eigenmodes, we use δ = 50 nm to cover the variance from the non-resonance contributions. *h* is computed as:

10 $$\begin{array}{*{20}c} {h = {\Gamma }_{{{\text{ex}}}} + {\Gamma }_{{{\text{em}}}} } \\ \end{array}$$

Here we define the eigenmode enhancement factors as Γ_ex_ and Γ_em_, which are the sum of the eigenmode intensity at each size normalized with the maximum intensity of all sizes:

11 $$\begin{array}{*{20}c} {{\Gamma }_{{{\text{ex}}}} = \frac{{\mathop \sum \nolimits_{j}^{{N_{a} }} {\varvec{E}}_{j}^{2} }}{{{\text{max}}\left( {\mathop \sum \nolimits_{j}^{{N_{a} }} {\varvec{E}}_{j}^{2} } \right)}}} \\ \end{array}$$

12 $$\begin{array}{*{20}c} {{\Gamma }_{{{\text{em}}}} = \frac{{\mathop \sum \nolimits_{k}^{{N_{a} }} {\varvec{E}}_{k}^{2} }}{{{\text{max}}\left( {\mathop \sum \nolimits_{k}^{{N_{a} }} {\varvec{E}}_{k}^{2} } \right)}}} \\ \end{array}$$

where Γ_ex_ is the excitation eigenmodes factor, Γ_em_ is the emission eigenmodes factor, *j, k* are the eigenmode number and *N*_*a*_ represents the total number of modes under pillar size *a*. *I*_0_ is the no-pillar intensity calculated according to Eqs. (1) and (2) in the previous section, and it represents the direct emission only related to the implanted area without resonance in the pillar. The weights in front of each term can possibly be variable, subject to different size scenarios, here we assume all weights as one for simplicity.

The envelop of the eigenmodes peaks appeared at 200 nm - 1 µm are illustrated in Fig. 6 a and b. The contribution from excitation and emission eigenmodes are shown in with the blue and red peaks respectively, and the gray area shows the contribution from implantation area without pillars. Some significant eigenmodes appeared at around 600 nm in both circular and square pillars, and this trend is in good agreement with the experimental results in Fig. 3, being verified the enhancement theory shown by Eq. (8). A Lorentz curve based on Eqs. (8) and (9) centered at 600 nm is used as the ultimate fitting to show the enhancement trend (green curves). For the circular pillars, the high resonance peak at 600 nm can also explain the two outliers in Fig. 3b with extreme high intensity. Figure 6c–d present the eigenmode pattern in circular pillars at the maximum enhancement size of λ_ex_ and λ_em_ respectively, and Fig. 6e–f present the eigenmodes in square pillars, which all clearly showing the feature of resonance modes.

## Conclusion

We fabricated Pr-implanted GaN nanopillars with different sizes, down to a minimum diameter and side-length of 100 nm for circular-shapes and square-shapes, and investigated their RT photon emission properties. It was confirmed by SEM observation that all pillars were fabricated at their desired size mostly within the error of 5%. We clarified using the home-built CFM that the implanted Pr ions in GaN showed two main emission peaks at 650.3 nm and 651.8 nm, which are attributed to ^3^*P*_0_-^3^*F*_2_ transition in the 4*f*-shell. We also observed resonant excitation peaks at 506 nm (2.45 eV) and 525 nm (2.36 eV), which are presumably due to the ^3^*H*_4_-^3^*P*_1_ transition. These optical transitions were unaffected by pillar structures. Dependences of the photon emission intensities on pillar sizes and excitation powers were systematically investigated and as a result, we found that the photon emisssion enhancement appeared when the size of nanopillars were less than 1 µm and the highest enhancement ratio was obtained from the 200 nm-sized circular pillar. The maximum value for the enhancement ratio was 23.5 when the excitation power density was lowest (0.67 kW/cm^2^). This value can be divided into the emitted photon extraction enhancement by a factor of 4.5 and the photon collection enhancement by a factor of 5.2, from the analysis of photon emission saturation behaviors. We established a theoretical model to analyze the enhancement based on coupling to the simulated eigenmodes at both the excitation and emission wavelenth. The change in enhancement ratio with different pillar sizes was explained by the envelope of those eigenmodes. Coupling to a series of eigenmodes generates broadband enhancement and the PL spectral shape of Pr ions remains unchanged by nanopillar structures. Our study paves the way for lanthanoid-doped GaN nano/micro-scale photon emitters and quantum technology applications, although optimizaiton of pillar structures and improvement of fabrication processes could be further explored for their realization.

## Supplementary Information

Supplementary Information.

## Data Availability

The datasets used and/or analyzed during the current study available from the corresponding author on reasonable request.
