# Supplementary material for: Photon extraction enhancement of praseodymium ions in gallium nitride nanopillars
Source: Sci Rep. 2022 Dec 8;12:21208. doi: 10.1038/s41598-022-25522-6 (PMC9731982; doi:10.1038/s41598-022-25522-6)
Supplement: Supplementary file 1 — Supplementary Information. [file 41598_2022_25522_MOESM1_ESM.pdf]

Supplementary Information

Photon extraction enhancement of praseodymium ions in gallium nitride nanopillars

Shin-ichiro Sato,[1,2*] Shuo Li,[2] Andrew D. Greentree,[2] Manato Deki,[3] Tomoaki Nishimura,[4] Hirotaka Watanabe,[5] Shugo Nitta,[5] Yoshio Honda,[5] Hiroshi Amano,[3,5] Brant C. Gibson,[2] and Takeshi Ohshima[1]

[1]*Quantum Beam Science Research Directorate, National Institutes for Quantum Science and Technology, 1233 Watanuki, Takasaki, Gunma 370-1292, JAPAN*

[2]*Australian Research Council Centre of Excellence for Nanoscale BioPhotonics, RMIT University, Melbourne, Victoria 3001, AUSTRARIA*

[3]*Venture Business Laboratory, Nagoya University, Furo-cho, Chikusa-ku, Nagoya, Nagoya 464-8601, JAPAN*

[4]*Research Center of Ion Beam Technology, Hosei University,3-7-2 Kajino-cho, Koganei, Tokyo 184-8584, JAPAN*

[5]*Institute of Materials and Systems for Sustainability, Nagoya University, Furo-cho, Chikusa-ku, Nagoya, Nagoya 464-8601, JAPAN*

Corresponding author: Shin-ichiro Sato (sato.shinichiro2@qst.go.jp)

Resonant excitation of Pr-doped GaN

To explore the detail of energy levels which contribute to the excitation, the photoluminescence excitation (PLE) of Pr-implanted GaN was investigated at RT. The sample measured was undoped GaN epilayer on Sapphire substrate implanted with 150 keV-Pr ions at the fluence of 1.0×10^{14} cm^{-2}. Thermal annealing at 1200 ^{o}C for 1 min under N_2 atmosphere was conducted after implantation. The Pr-implanted area was 1×1 μm^2 square regions and the background was subtracted to remove the effect of photon emission from unwanted defects. The result is shown in Fig. S1. First the wide range scan from 400 nm to 610 nm revealed that the high intensity photon emission appeared when excited through around 400-440 nm and 500-540 nm due to the resonant excitation of Pr^{3+} ions. The excitation wavelength was then scanned in 1 nm steps and two pronounced peaks were observed with emission at 506 nm (2.45 eV) and 525 nm (2.36 eV). The PL intensity increased with reducing the excitation wavelength at below 440 nm. According to the Dieke's diagram, the peaks at 506 nm and 525 nm are thought to be due to the transition from $^3H_4$ state to $^3P_1$ state[1,2], although further investigation is required for more accurate assignment. We chose to excite with a wavelength at 525 nm (2.36 eV) for efficient Pr^{3+} excitation throughout this study (Figs. 2, 3, and 4 in the main text), except where otherwise noted.

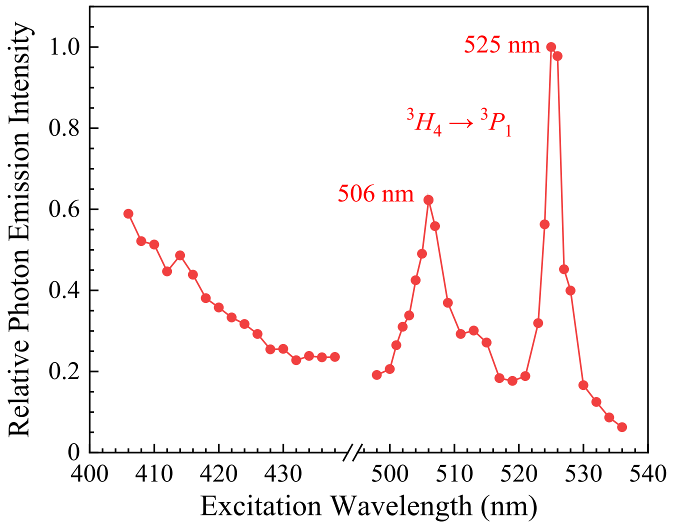

Fig. S1. PLE spectrum of 1 μm×1 μm Pr-implanted GaN where no pillars were etched at RT. The bandwidth of excitation laser was approximately 1 nm.

References

1 Birkhahn, R., Garter, M. & Steckl, A. J. Red light emission by photoluminescence and electroluminescence from Pr-doped GaN on Si substrates. *Appl. Phys. Lett.* **74**, 2161-2163, doi:10.1063/1.123787 (1999).

2 Lozykowski, H. J., Jadwisienczak, W. M. & Brown, I. Photoluminescence and cathodoluminescence of GaN doped with Pr. *J. Appl. Phys.* **88**, 210-222, doi:10.1063/1.373645 (2000).
